# Voltage Sensor Gating Charge Transfer in a hERG Potassium Channel Model

**DOI:** 10.1016/j.bpj.2014.10.001

**Published:** 2014-11-18

**Authors:** Charlotte K. Colenso, Yang Cao, Richard B. Sessions, Jules C. Hancox, Christopher E. Dempsey

**Affiliations:** 1 School of Biochemistry, University of Bristol, Bristol, United Kingdom; 2 School of Physiology and Pharmacology, University of Bristol, Bristol, United Kingdom

## Abstract

Relaxation of a hERG K^+^ channel model during molecular-dynamics simulation in a hydrated POPC bilayer was accompanied by transitions of an arginine gating charge across a charge transfer center in two voltage sensor domains. Inspection of the passage of arginine side chains across the charge transfer center suggests that the unique hydration properties of the arginine guanidine cation facilitates charge transfer during voltage sensor responses to changes in membrane potential, and underlies the preference of Arg over Lys as a mobile charge carrier in voltage-sensitive ion channels.

The response of voltage-sensitive ion channels to changes in membrane potential is mediated by voltage sensor domains (VSD) containing a transmembrane helical segment (S4) with a repeating motif of positively charged and hydrophobic amino acids (Fig. 1) (1,2). Changes in membrane potential drive the S4 helix through the membrane plane with the charged side chains (largely arginine) on S4 swapping Glu/Asp carboxylate partners that lie on less mobile elements of the VSD (2). Movement of S4 is coupled to the ion-conducting pore to transmit changes in membrane potential to channel gating (3).

The VSD charge-pairing motif of K^+^ and Na^+^ channels is best represented in VSD states at zero membrane potential (S4 helix up) for which crystal structures exist for Kv1.2 (4), Kv1.2/2.1 chimera (5), and Na_v_ channels (6,7). In these states, positively charged residues on the intra- and extracellular sections of the S4 helix are separated by a hydrophobic charge-transfer center (CTC) (1) or plug (8) containing a highly conserved Phe residue (Fig. 1). This plug restricts water incursion across the VSD, focusing the electric field across a narrow region near the bilayer center. In voltage-driven transitions between S4 down- and up-states, positively charged S4 side chains move across the CTC.

The ether-à-go-go (eag) and eag-related family of voltage-sensitive K^+^ channels likely share similar charge pairing interactions with VSDs in other channels (9,10). However, eag VSDs contain an extra negative charge on S2 (underlined in Fig. 1 *C*) so that in hERG, Asp residues (D460 and D466) lie approximately one helical turn above and below the conserved charge-transfer center Phe (F463) (Fig. 1). This eag-specific motif might be expected to facilitate transfer of Arg side chains through the CTC and to stabilize the voltage sensor (VS) in the up state. We recently described an open state (VS-up) hERG model built on the crystal structure template of the Kv1.2/2.1 chimera and molecular-dynamics (MD) simulation of this model in a hydrated POPC bilayer (11). We have inspected an extended version of this simulation and identified transitions of a gating charge into the CTC despite the absence of a membrane potential change. These transitions are absent in equivalent MD simulations of the chimera structure in a POPC bilayer.

Fig. 1 shows a single VS from starting structures of the hERG model and the chimera structure in a hydrated POPC bilayer, after restrained MD to anneal the protein-lipid interface (see Methods in the Supporting Material). Because the hERG model is constructed on the chimera structure according to the alignment in Fig. 1 the pattern of pairing between S4 charges and acidic VS side chains is equivalent in the hERG model and chimera structure.

The arrangement of charge-paired side chains remains constant during MD in all subunits of the chimera (e.g., Fig. 2 *E* and see Fig. S2 in the Supporting Material). However, in two subunits of the hERG model the R534 side chain moves toward the extracellular side of the bilayer, sliding into the CTC to form a charge interaction with the extra Asp residue (D460 in hERG) that lies just above F463 (Fig. 2, *A*–*C*). This transition is facilitated by changes in side-chain rotamers of R534 and F463 as the planar Arg guanidine group rotates past the F463 ring, and the availability of D460 as a counterion for the R534 guanidine (Fig. 2). Movement of an Arg guanidine past the Phe side chain of the CTC is similar to that described in steered MD of an isolated VS domain (12).

Mason et al. (13) have shown, using neutron scattering, that the low charge density guanidine cation (Gdm^+^) corresponding to the Arg side chain is poorly hydrated above and below the molecular plane. This property may underlie the universal preference for Arg (over Lys) in voltage sensor charge transfer. Although the poorly-hydrated surfaces of Gdm^+^ interact favorably with nonpolar (especially planar) surfaces (14,15), Gdm^+^ retains in-plane hydrogen bonding (13). In the transition of R534 across the CTC, in-plane solvation of the guanidine side chain is provided initially by D466, D501, and water molecules below the CTC, and during and after the transition by D501 and D460 side chains and waters above the CTC (Fig. 3, *A* and *B*). Complete transfer of the R534 side chain across the CTC was not observed, but would be expected to involve movement of the guanidine group away from H-bonding distance with D501.

The atom distribution around the R534 side chain during MD (Fig. 3, *B* and *C*) conforms to the experimental Gdm^+^ hydration structure (13), with H-bonding to waters and side-chain Asp O atoms exclusively in the guanidine plane. The passage of Gdm^+^ through the CTC is facilitated by the hydrophobic nature of Gdm^+^ above and below the molecular plane (13), which allows interaction with the nonpolar groups (especially F463) in the CTC (Fig. 3 *A* and see Fig. S3). This contrasts with the solvation properties of the Lys amino group (e.g., K302 of the Kv1.2/2.1 chimera (Fig. 1), which has a spherical distribution of H-bonding and charge-neutralizing oxygen atoms (Fig. 3 *D* and see Fig. S4).

To further test these interpretations, we ran additional MD simulations of the isolated hERG VS domain model and an R534K mutant in a hydrated POPC bilayer. Again, the R534 side chain entered the CTC in the wild-type model simulation whereas the K534 side chain did not (see Fig. S5). Inspection of the atom distributions in Fig. 3 *D* (and see Fig. S4) indicates that the pocket below the conserved Phe of the CTC is particularly favorable for a Lys side chain, with waters and acidic side chains that satisfy the spherical solvation requirements of the terminal amino group, and nonpolar side chains that interact with the aliphatic part of the side chain.

The occurrence of transitions of the R534 side chain through the CTC in the hERG model, in the absence of a change in membrane potential, indicates a relaxation from a less-stable starting structure. However, the path of the R534 side chain provides useful molecular-level insight into the nature of charge transfer in voltage sensors. How do these observations accord with broader evidence of charge transfer in voltage-sensitive channels in general, and hERG in particular? Studies with fluorinated analogs of aromatic side chains equivalent to F463 of hERG or F233 of the chimera indicate the absence of a significant role for cation-*π* interactions involving the CTC aromatic group in K^+^ and Na_v_ channels, although a planar side chain is preferred in some cases (1,16). In hERG, F463 can be replaced by M, L, or V with small effects on channel gating (17), indicating that the hERG CTC requires only a bulky nonpolar side chain to seal the hydrophobic center of the VS and allow passage of the Arg side chain through the CTC. Both absence of requirement for cation-*π* interactions, and accommodation of nonplanar hydrophobic side chains in a functional hERG CTC, are broadly consistent with the interpretation that it is the poorly-hydrated nature of the Arg guanidine group above and below the molecular plane (together with its tenacious proton affinity (18)) that governs its role in carrying gating charge in voltage sensors.

While the simulations suggest that R534 may interact with D460 in the open channel state, the possibility that the extra carboxylate side chain above the CTC might facilitate gating charge transfer is seemingly inconsistent with the slow activation of hERG, although hERG D460C does activate even more slowly than the WT channel (9). However, S4 movement in hERG occurs in advance of channel opening (19), and slow gating is partly mediated by interactions involving hERG cytoplasmic domains (20); thus, slow S4 movement may not be an inherent property of the hERG voltage sensor. Recent studies show that when hERG gating is studied at very low [Ca^2+^] (50 *μ*M) and low [H^+^] (pH 8.0), the channel is strongly sensitized in the direction of the open state; this effect is reduced in hERG D460C (and hERG D509C) (10). These observations support a role for the extra hERG Asp residues in binding Ca^2+^ (and H^+^) (10), allowing the channel to be allosterically responsive to changes in pH and [Ca^2+^]. A true comparison of a hERG model with experimental channel gating might involve studies on a channel lacking cytoplasmic domains that modulate gating, and using conditions (high pH and low [Ca^2+^]) that leave the eag-specific Asp residues unoccupied. This could reveal the inherent current-voltage relationships and kinetics of the hERG voltage sensor.

## Figures and Tables

**Figure 1:**
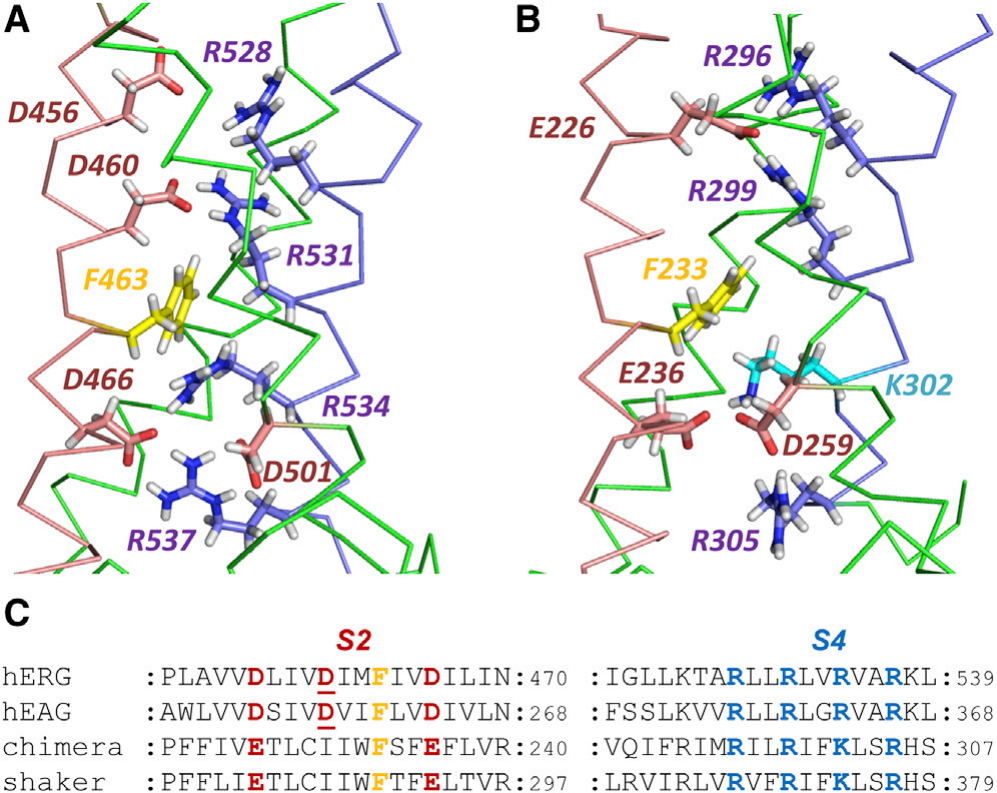
Structures of the VSD of membrane domains before MD in a POPC bilayer. The S2 (*pink*) and S4 (*blue*) helices of the VSD of the hERG model (*A*) and Kv1.2/2.1 chimera structure (*B*) are highlighted. (*C*) Sequence alignment of S2 and S4 among homologous voltage-sensitive K^+^ channels.

**Figure 2:**
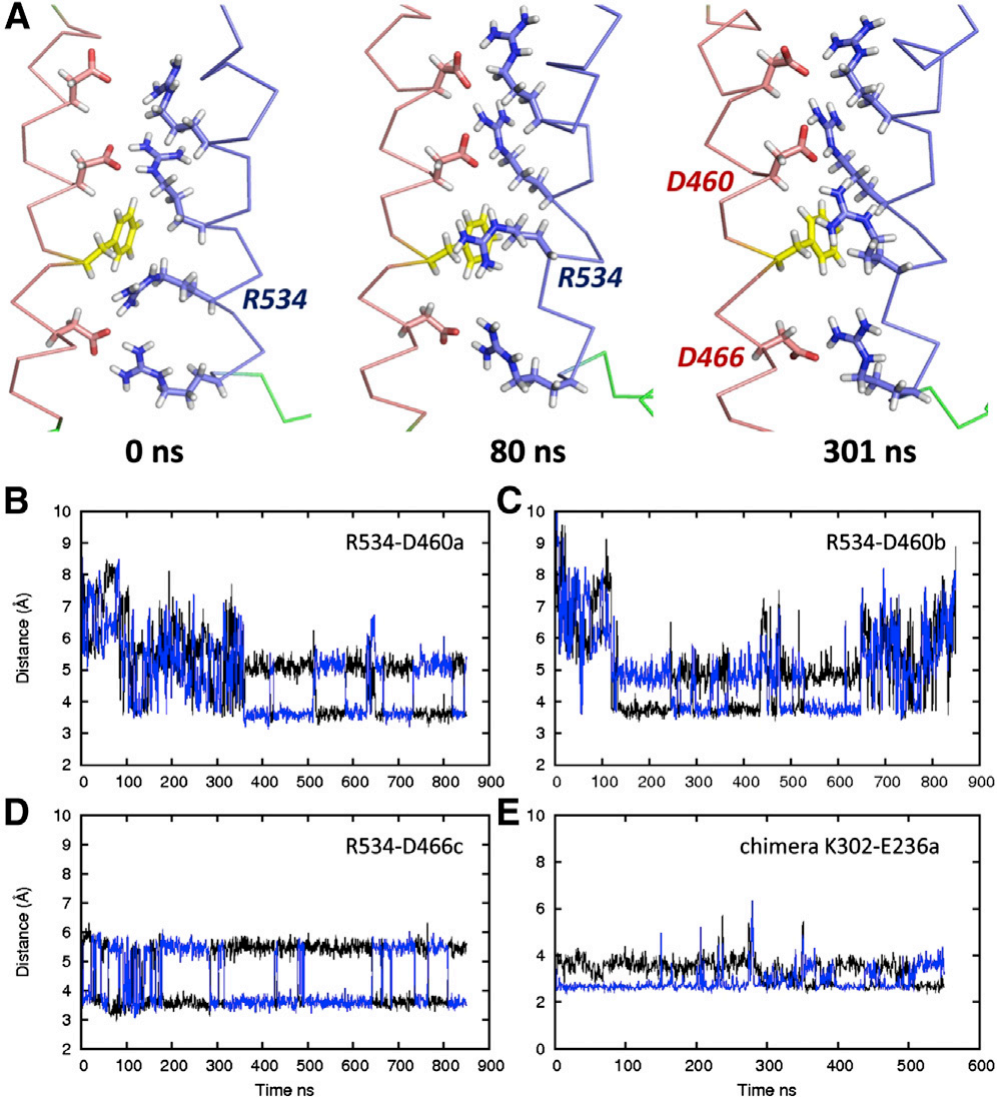
Movement of the R534 side chain across the CTC in chain *a* of the hERG model simulation (*A*). Similar transitions are observed in chains *a* and *b* (panels *B* and *C*), but not chains *c* (*D*) or *d* (not shown), where the R534 side chain remains close to D466. In all subunits of the Kv1.2/2.1 chimera simulation, charge pairing of the starting structure (Fig. 1*B*) was maintained throughout (e.g., panel *E* and see Fig. S2 in the Supporting Material). (*Black* and *blue lines*) Distances from the Arg CZ or Lys *ε* atom to the two O atoms, respectively, of Asp or Glu.

**Figure 3:**
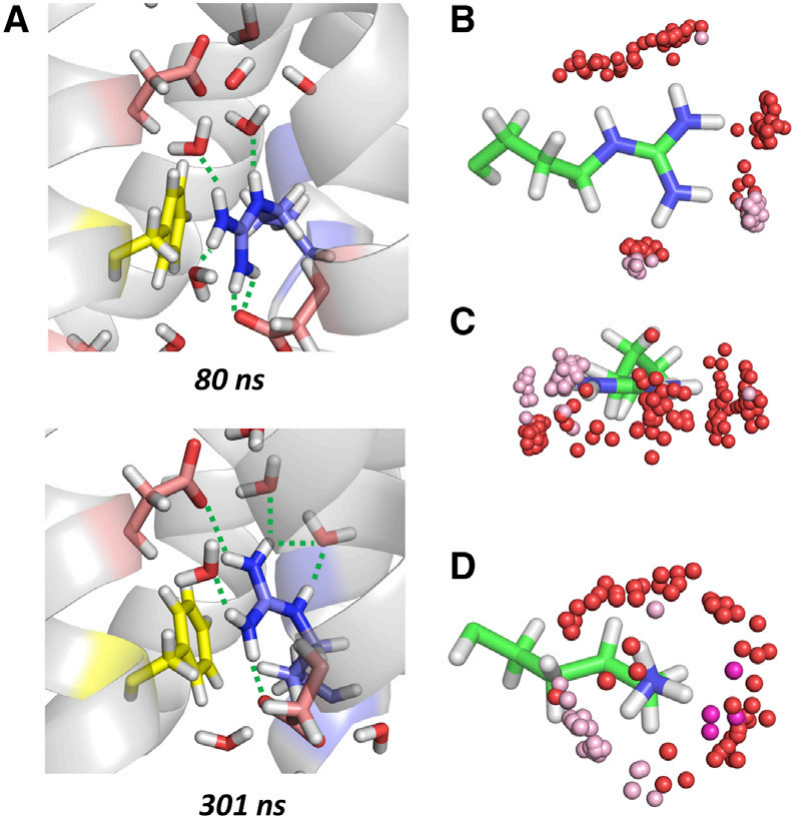
In-plane solvation of R534 guanidine in the charge transfer center during the hERG model MD (*A*). (*Dotted lines*) H-bond distances of <2.5 Å. The right-hand group consists of top-down (*B*) and end-on (*C*) views of the distribution of oxygen atoms around the side chain of hERG R534 at 20-ns intervals during MD (subunit *a*). (*D*) End-on view of equivalent atom distributions around the K302 side chain during the Kv1.2/2.1 chimera MD (subunit *c*). (*Red spheres*, water O; *pink*, Asp OD1 and OD2; *purple*:, Glu OE1 and OE2.)

## Supporting Material

Document S1. Additional supplemental information, methods, and five figures

Document S2. Article plus Supporting Material

## Supporting Citations

References (21–27) appear in the Supporting Material.
