# Supplementary material for: Voltage Sensor Gating Charge Transfer in a hERG Potassium Channel Model
Source: Biophys J. 2014 Nov 18;107(10):L25–8. doi: 10.1016/j.bpj.2014.10.001 (PMC4241455; doi:10.1016/j.bpj.2014.10.001)
Supplement: Document S1. Additional supplemental information, methods, and five figures [file mmc1.pdf]

Voltage sensor gating charge transfer in a hERG potassium channel model

Charlotte K. Colenso,* Yang Cao,* Richard B. Sessions,*Jules C. Hancox,† and Christopher E. Dempsey*
*School of Biochemistry, and † School of Physiology and Pharmacology, University of Bristol, Bristol BS8 1TD, UK.

Supporting Material

Methods

Molecular dynamics simulation: The molecular dynamics (MD) simulation of the hERG membrane domain model in a hydrated POPC bilayer at 310 K over 0.5 μs has been described (1). The analyses here and in the main paper were made on this simulation extended to 0.85 μs.

Gromacs 4.5.3 (2) was used for all MD set up and simulation runs. We made two equivalent 0.5 – 0.55 μs MD simulations of the Kv1.2/2.1 chimera structure (the template for the hERG model) in hydrated POPC membrane patches at 310 K, one incorporating only the membrane (α) domain of the channel (0.55 μs) and one containing both the α- and cytoplasmic domains (0.50 μs). Briefly, the crystal structure coordinates (PDB: 2R9R) (3) were downloaded and the cytoplasmic (T1) domain either retained ("full") or removed ("alpha"). Two of the K^+ ions in the selectivity filter were replaced with water molecules to yield a 0101 (K^+ = 1; water = 0) occupancy state as counted from the extracellular side of the channel. The channels were energy minimized with 1800 steps of steepest descent and conjugate gradient methods using Discover within InsightII (Accelrys; San Diego, CA, USA) and were then embedded in a POPC bilayer consisting of 576 lipid molecules in each bilayer leaflet using g-membed (4), before solvation with a 15 Å layer of water above and below the membrane. The water solution contained a number of K^+ and Cl^- ions adjusted to yield a concentration of 140 mM and to maintain overall charge-neutrality.

The simulation systems were energy minimized using 2000 steps of steepest descent and were then heated to 310 K for 5 ns of MD during which the non-hydrogen protein atoms and selectivity filter K^+ ions were restrained. Production runs were then performed for 0.85 μs (hERG model) or 0.50 - 0.55 μs (chimera models) using the OPLS all-atom forcefield (5), Berger lipid parameters (6), and the SPC water model (7). All other simulation parameters were as described in (1).

Isolated single voltage sensor simulations were made by editing the hERG model coordinate file to leave only a single voltage sensor (subunit a) which was embedded in a reduced POPC bilayer (256 lipids in each monolayer). A hERG voltage sensor R534K mutant was made by changing the R534 side chain to K534 having the same side chain rotamer as K302 in the Kv1.2/2.1 chimera structure. The systems were solvated with water and ions, energy minimized and subjected to 5 ns restrained dynamics as described above, before 200 ns production MD runs at 310 K.

Supporting Figures

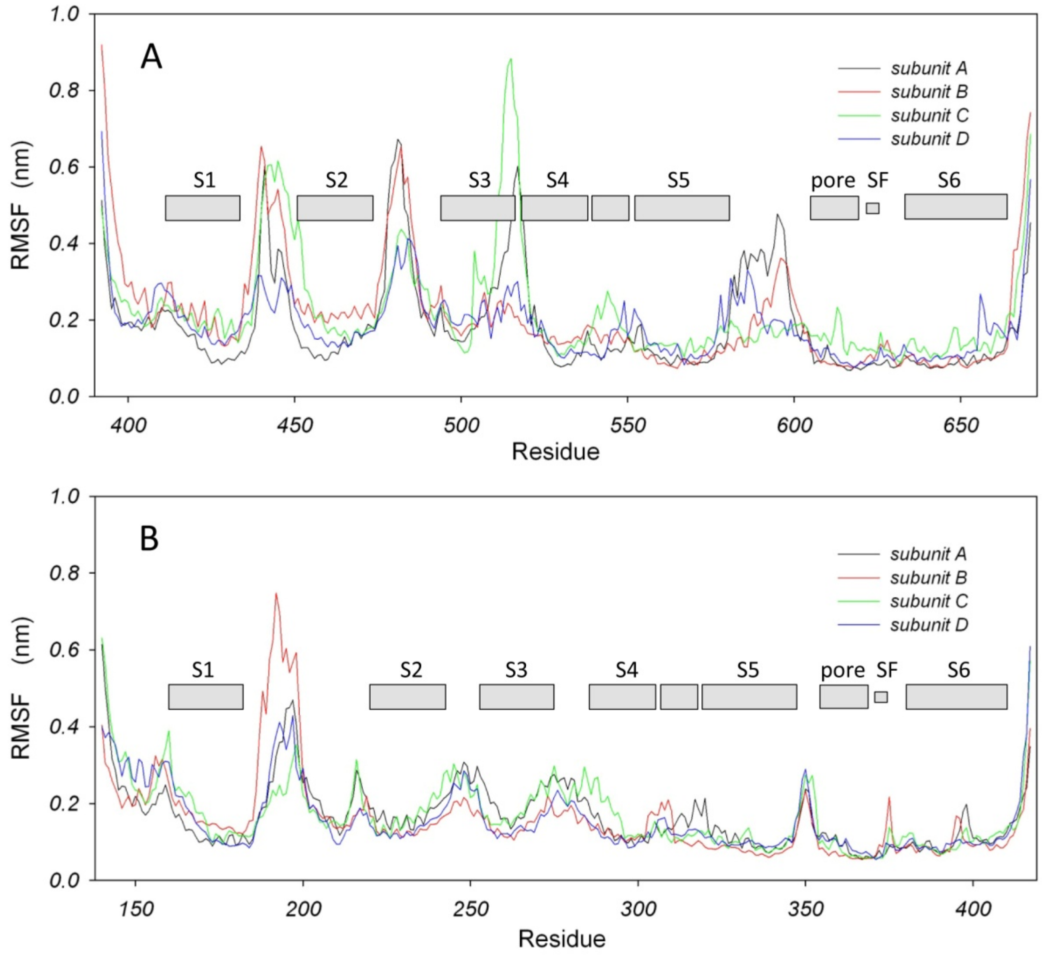

Figure S1 Root mean square fluctuation (RMSF) for Cα atoms of the hERG (A) and Kv1.2/2.1 chimera (B) membrane domain simulations in hydrated POPC membranes at 310 K. The profile of RMSF values for the chimera simulation is very similar to that described by Bjelkmar et al. (8) for a closely related (Kv1.2) K^+ channel simulation in a POPC/POPG mixed membrane at 300 K. SF = selectivity filter.

As previously found for MD simulations of membrane incorporated proteins, extramembrane loops connecting transmembrane (TM) elements often show high conformational flexibility (high RMSF) during MD (*e.g.* ref 8). This applies to the long extracellular loop between S1 and S2 in the chimera simulation (Fig. S1 B), and the extracellular loops between S1 and S2, and between S5 and the pore helix, and the cytoplasmic loop between S2 and S3 in hERG (Fig. S1 A).

The extracellular loop between S3 and S4 is truncated in hERG compared to the chimera template and this region is difficult to model onto the template chimera structure (1). Although two subunits (a and c corresponding to the black and green traces in Fig. S1 A) showed high RMSF for the C-terminal end of S3 and its linker with S4, this high flexibility did not directly affect the C-terminal end of S4 and the charge transfer centre (CTC) discussed in the main text. Of the two hERG VS subunits showing movement of the R534 side chain across the CTC (subunits a and b; see main text), one showed high RMSF in the S3-S4 linker (subunit a) and one showed low RMSF in this region (subunit b; see Fig. S1A).

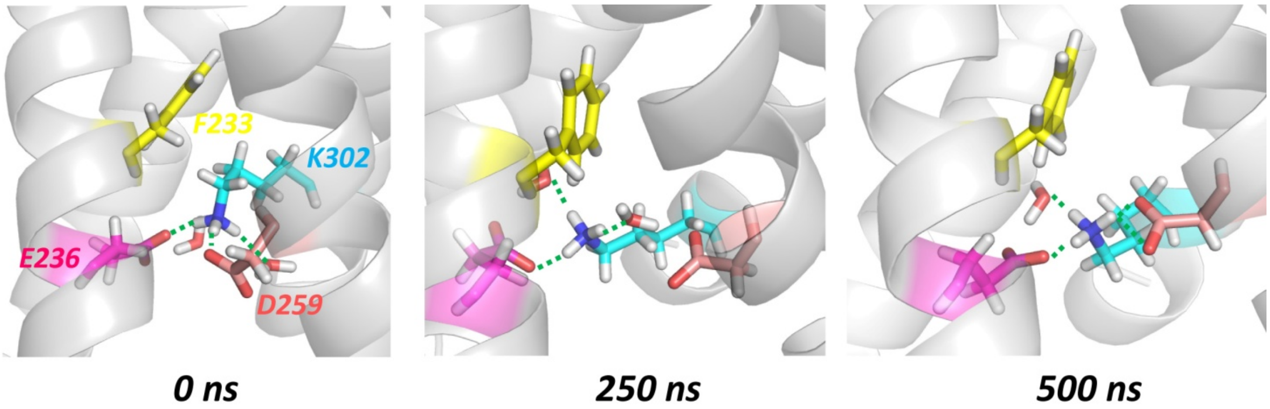

Figure S2 "Solvation" of the K302 amino group during MD simulation of the Kv1.2/2.1 chimera membrane domain. Dotted lines denote hydrogen bond distances of less than 2.5 Å.

The K302 amino group of the Kv1.2/2.1 chimera (see Fig. 1 of main text) maintained hydrogen bond interactions with carboxylate side chains and water molecules below F233 of the charge transfer centre (CTC) throughout 550 ns of MD despite fluctuations around a generally stable structure. In the example shown (subunit a) fluctuations temporarily move the K302 amino group from hydrogen bond interaction with the D259 carboxylate (e.g. at 250 ns). The stability of the Kv1.2/2.1 chimera starting structure was maintained in all 4 subunits of each of the simulations in hydrated POPC membranes in which the cytoplasmic subunit was either retained or removed (> 4 μs total simulation time).

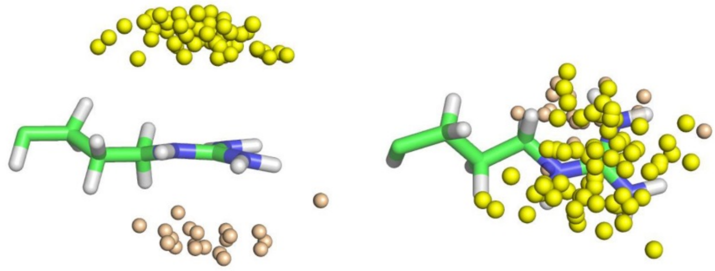

Figure S3 Distribution of non-polar carbon atoms around the R534 side chain of the WT hERG simulation as the guanidine ring moves past F463 of the CTC. Yellow spheres are side chain carbons of F463 within 4.5 Å of the guanidine CZ atom at 20 ns intervals in the first 200 ns of MD of subunits a and b (see Fig. 2 A-C main paper). Wheat-colored atoms are other hydrophobic carbons within 4.5 Å of the guanidine CZ. Left: side view; right: top-down view.

The solvation-deficient faces of the guanidine side chain (see Fig. 3 B,C of main paper) can interact with solvation-deficient surfaces of non-polar amino acids as previously described (9, 10). In this case the R534 side chain slides between the F463 side chain (yellow spheres in Fig. S3) and other non-polar amino acids (largely side chain carbons of I500 and A504 on the S3 helix).

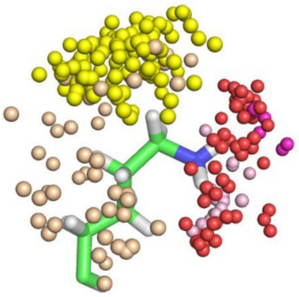

Figure S4 Atom distributions around the K302 side chain of the Kv1.2/2.1 chimera VS (see Fig. 1 and Fig. S2) at 20 ns intervals during MD (20-540 ns; subunit c). Yellow spheres: F233 side chain carbons; wheat: other non-polar C atoms; red: water O atoms; pink: Asp carboxylate O atoms; purple: Glu carboxylate O atoms. This is the same compilation as shown in Fig. 3D of the main paper but rotated 180 ^{o} around z and y (x,y is in the plane of the page), and with non-polar C-atoms within 4.5 A of Cδ, Cε and Nz added.

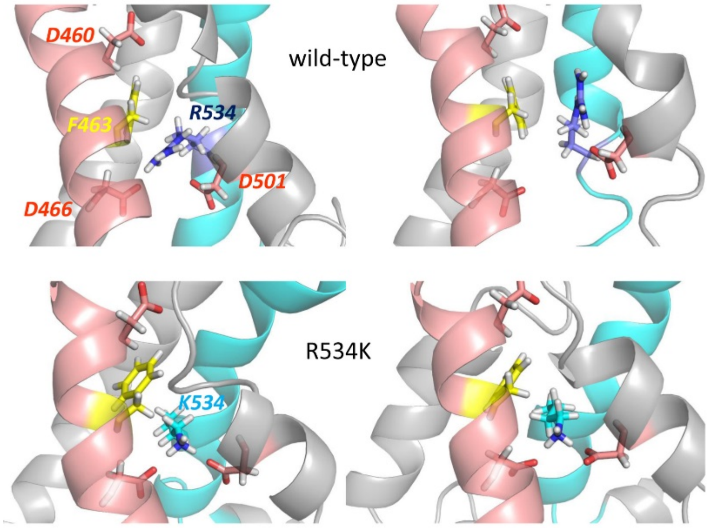

Figure S5 Starting (left) and final (200 ns; right) conformation of isolated hERG wild-type (top) and R534K model (bottom) voltage sensor simulations in a solvated POPC bilayer patch at 310 K. The simulations were run as described under Methods.

The cavity below the conserved Phe residue (F233 of the Kv1.2/2.1 chimera; F463 of hERG) may constitute a “trap” for the Lys side chain. As indicated in the Fig. S2 legend, the K302 side chain of the chimera simulation did not move from this cavity during a total of 4 μs of MD (4 subunits each of the chimera simulation with and without the cytoplasmic domain). Fig. S4 illustrates that the environment of this cavity conforms to the heterogeneous nature of the Lys side chain with non-polar interactions between chimera residues (F233: yellow spheres; I173, A262 and I263: wheat spheres in Fig. S4) and the non-polar part of the K302 side chain, with waters and carboxylate O atoms of E236 and D259 solvating the charge focused on the end of the side chain.

These interpretations are supported by additional simulations of the isolated wild-type hERG VS domain and an R534K “mutant” in a hydrated POPC bilayer. The R534 side chain guanidine moved into the non-polar gap of the CTC in the wild-type simulation (Fig. S5 top), while the K534 side chain of the mutant remained fixed in the cavity below the F463 side chain of the CTC. An additional 200 ns R534K mutant run in which the mutant VS was independently re-embedded in the POPC bilayer before MD also resulted in a stable retention of the K534 side chain in the pocket below F463 (not shown).

We note that the hERG membrane domain open channel model of Durdagi et al. (11) has a similar set of VS charge interactions as in our wild-type hERG model. However MD simulations in that study were of 7.5

ns duration, limiting the possibility of observing transitions on the ~ 100 ns timescale and precluding direct comparison with the VS transitions described here.

Supporting References

1. Colenso, C. K., R. B. Sessions, Y.-H. Zhang, J. C. Hancox and C. E. Dempsey. 2013. Interactions between voltage sensor and pore domain in a hERG K^+ channel model from molecular simulations and the effects of a voltage sensor mutation. *J. Chem. Inf. Model.* 53: 1358-1370.

2. Hess, B., C. Kutzner, D. van der Spoel and E. Lindahl. 2008. GROMACS 4: Algorithms for highly efficient, load-balanced, and scalable molecular simulations. *J. Chem. Theory Comput.* 4: 435-447.

3. Long, S. B., X. Tao, E. B. Campbell and R. Mackinnon. 2007. Atomic structure of a voltage-dependent K+ channel in a lipid membrane-like environment. *Nature* 450: 376-382.

4. Wolf, M. G., M. Hoefling, C. Aponte-Santamaria, H. Grubmuller and G. Groenhof. 2010. g_membed: Efficient insertion of a membrane protein into an equilibrated lipid bilayer with minimal perturbation*. J. Comput. Chem.* 31: 2169-2174.

5. Jorgensen W. L. and J. Tirado-Rives. 1988. The OPLS potential function for proteins – energy minimizations for crystals of cyclic-peptides and crambin. *J. Am. Chem. Soc.* **110**, 1657-1666.

6. Berger, O., O. Edholm and F. Jahnig. 1997. Molecular dynamics simulations of a fluid bilayer of dipalmitoylphosphatidylcholine at full hydration, constant pressure, and constant temperature. *Biophys. J.* **72**, 2002-2013.

7. Berendsen, H., J. P. M. Postma, W. F. van Gunsteren and J. Hermans. 1981. Interaction models for water in relation to protein hydration. In *Intermolecular Forces*; Pullman, B. Ed.; Reidel: Dordrecht; pp 331-342.

8. Bjelkmar, P., P. S. Niemelä, I. Vattulainen and E. Lindahl. 2009. Conformational changes and slow dynamics through microsecond polarized atomistic molecular simulation of an integral Kv1.2 ion channel. *PLoS Comp. Biol.* e1000289.

9. Mason, P. E., J. W. Brady, G. W. Neilson and C. E. Dempsey. 2007. The interaction of guanidinium ions with a model peptide. *Biophys. J.* 93: L04-L06.

10. Mason, P. E., C. E. Dempsey, G. W. Neilson, S. R. Kline and J. W. Brady. 2009. Preferential interaction of guanidinium ions with aromatic groups over aliphatic groups. *J. Am. Chem. Soc.* 131: 16680-16696.

11. Durdagi, S., S. Deshpande, H. J. Duff and S. Y. Noskov. 2012. Modeling of open, closed and open-inactivated states of the hERG1 channel: structural mechanisms of the state-dependence of drug binding. *J. Chem. Inf. Model.* 52: 2760-2774.
