# Supplementary material for: Voltage Sensor Gating Charge Transfer in a hERG Potassium Channel Model
Source: Biophys J. 2014 Nov 18;107(10):L25–8. doi: 10.1016/j.bpj.2014.10.001 (PMC4241455; doi:10.1016/j.bpj.2014.10.001)
Supplement: Document S2. Article plus Supporting Material [file mmc2.pdf]

Biophysical Letter

Voltage Sensor Gating Charge Transfer in a hERG Potassium Channel Model

Charlotte K. Colenso,[1] Yang Cao,[1] Richard B. Sessions,[1] Jules C. Hancox,[2] and Christopher E. Dempsey[1,*]
[1]School of Biochemistry and [2]School of Physiology and Pharmacology, University of Bristol, Bristol, United Kingdom

ABSTRACT Relaxation of a hERG K^+ channel model during molecular-dynamics simulation in a hydrated POPC bilayer was accompanied by transitions of an arginine gating charge across a charge transfer center in two voltage sensor domains. Inspection of the passage of arginine side chains across the charge transfer center suggests that the unique hydration properties of the arginine guanidine cation facilitates charge transfer during voltage sensor responses to changes in membrane potential, and underlies the preference of Arg over Lys as a mobile charge carrier in voltage-sensitive ion channels.

Received for publication 9 June 2014 and in final form 2 October 2014.

*Correspondence: c.dempsey@bristol.ac.uk

Charlotte Colenso's present address is Universidad Austral de Chile, Facultad de Medicina, Instituto de Fisiologia, Campus Isla Teja, Valdivia, 511-0566, Chile.

This is an open access article under the CC BY license (http://creativecommons.org/licenses/by/3.0/).

The response of voltage-sensitive ion channels to changes in membrane potential is mediated by voltage sensor domains (VSD) containing a transmembrane helical segment (S4) with a repeating motif of positively charged and hydrophobic amino acids (Fig. 1) (1,2). Changes in membrane potential drive the S4 helix through the membrane plane with the charged side chains (largely arginine) on S4 swapping Glu/Asp carboxylate partners that lie on less mobile elements of the VSD (2). Movement of S4 is coupled to the ion-conducting pore to transmit changes in membrane potential to channel gating (3).

The VSD charge-pairing motif of K^+ and Na^+ channels is best represented in VSD states at zero membrane potential (S4 helix up) for which crystal structures exist for Kv1.2 (4), Kv1.2/2.1 chimera (5), and Na_v channels (6,7). In these states, positively charged residues on the intra- and extracellular sections of the S4 helix are separated by a hydrophobic charge-transfer center (CTC) (1) or plug (8) containing a highly conserved Phe residue (Fig. 1). This plug restricts water incursion across the VSD, focusing the electric field across a narrow region near the bilayer center. In voltage-driven transitions between S4 down- and up-states, positively charged S4 side chains move across the CTC.

The ether-à-go-go (eag) and eag-related family of voltage-sensitive K^+ channels likely share similar charge pairing interactions with VSDs in other channels (9,10). However, eag VSDs contain an extra negative charge on S2 (underlined in Fig. 1 *C*) so that in hERG, Asp residues (D460 and D466) lie approximately one helical turn above and below the conserved charge-transfer center Phe (F463) (Fig. 1). This eag-specific motif might be expected to facilitate transfer of Arg side chains through the CTC and to stabilize the voltage sensor (VS) in the up state. We recently described an open state (VS-up) hERG model built on the crystal structure template of the Kv1.2/2.1 chimera and molecular-dynamics (MD) simulation of this model in a hydrated POPC bilayer (11). We have inspected an extended version of this simulation and identified transitions of a gating charge into the CTC despite the absence of a membrane potential change. These transitions are absent in equivalent MD simulations of the chimera structure in a POPC bilayer.

Fig. 1 shows a single VS from starting structures of the hERG model and the chimera structure in a hydrated POPC bilayer, after restrained MD to anneal the protein-lipid interface (see Methods in the Supporting Material). Because the hERG model is constructed on the chimera structure according to the alignment in Fig. 1 the pattern of pairing between S4 charges and acidic VS side chains is equivalent in the hERG model and chimera structure.

The arrangement of charge-paired side chains remains constant during MD in all subunits of the chimera (e.g., Fig. 2 *E* and see Fig. S2 in the Supporting Material). However, in two subunits of the hERG model the R534 side chain moves toward the extracellular side of the bilayer, sliding into the CTC to form a charge interaction with the extra Asp residue (D460 in hERG) that lies just above

Editor: Carmen Domene.
© 2014 The Authors
http://dx.doi.org/10.1016/j.bpj.2014.10.001

CrossMark

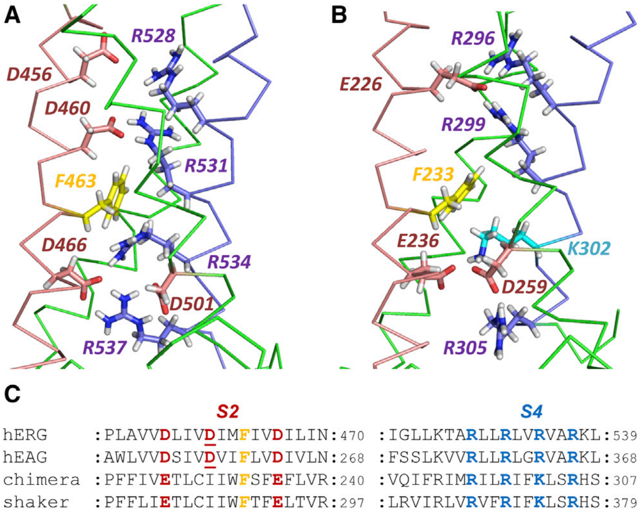

FIGURE 1 **Structures of the VSD of membrane domains before MD in a POPC bilayer. The S2 (*pink*) and S4 (*blue*) helices of the VSD of the hERG model (*A*) and Kv1.2/2.1 chimera structure (*B*) are highlighted. (*C*) Sequence alignment of S2 and S4 among homologous voltage-sensitive K$^+$ channels.**

F463 (Fig. 2, *A*–*C*). This transition is facilitated by changes in side-chain rotamers of R534 and F463 as the planar Arg guanidine group rotates past the F463 ring, and the availability of D460 as a counterion for the R534 guanidine (Fig. 2). Movement of an Arg guanidine past the Phe side chain of the CTC is similar to that described in steered MD of an isolated VS domain (12).

Mason et al. (13) have shown, using neutron scattering, that the low charge density guanidine cation (Gdm$^+$) corresponding to the Arg side chain is poorly hydrated above and below the molecular plane. This property may underlie the universal preference for Arg (over Lys) in voltage sensor charge transfer. Although the poorly-hydrated surfaces of Gdm$^+$ interact favorably with nonpolar (especially planar) surfaces (14,15), Gdm$^+$ retains in-plane hydrogen bonding (13). In the transition of R534 across the CTC, in-plane solvation of the guanidine side chain is provided initially by D466, D501, and water molecules below the CTC, and during and after the transition by D501 and D460 side chains and waters above the CTC (Fig. 3, *A* and *B*). Complete transfer of the R534 side chain across the CTC was not observed, but would be expected to involve movement of the guanidine group away from H-bonding distance with D501.

The atom distribution around the R534 side chain during MD (Fig. 3, *B* and *C*) conforms to the experimental Gdm$^+$ hydration structure (13), with H-bonding to waters and side-chain Asp O atoms exclusively in the guanidine plane. The passage of Gdm$^+$ through the CTC is facilitated by the hydrophobic nature of Gdm$^+$ above and below the molecular plane (13), which allows interaction with the nonpolar groups (especially F463) in the CTC (Fig. 3 *A* and see Fig. S3). This contrasts with the solvation properties of

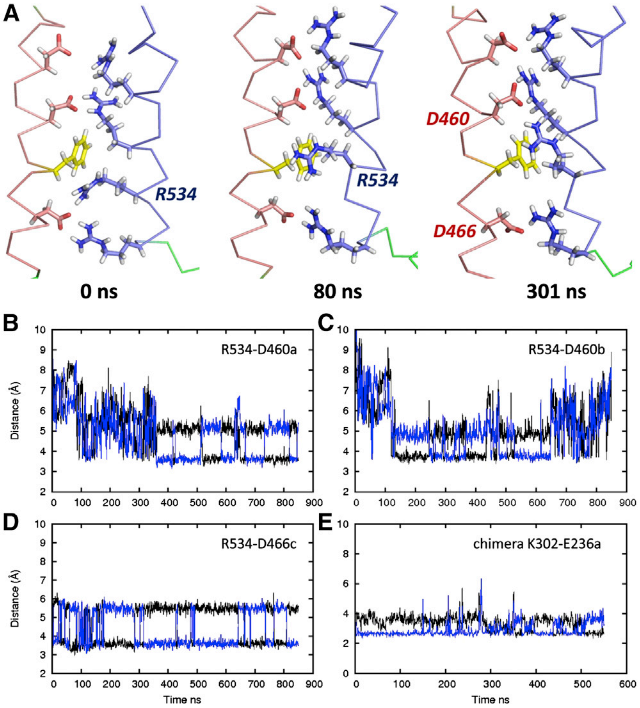

FIGURE 2 **Movement of the R534 side chain across the CTC in chain *a* of the hERG model simulation (*A*). Similar transitions are observed in chains *a* and *b* (panels *B* and *C*), but not chains *c* (*D*) or *d* (not shown), where the R534 side chain remains close to D466. In all subunits of the Kv1.2/2.1 chimera simulation, charge pairing of the starting structure (Fig. 1 *B*) was maintained throughout (e.g., panel *E* and see Fig. S2 in the Supporting Material). (*Black* and *blue lines*) Distances from the Arg CZ or Lys ε atom to the two O atoms, respectively, of Asp or Glu.**

the Lys amino group (e.g., K302 of the Kv1.2/2.1 chimera (Fig. 1), which has a spherical distribution of H-bonding and charge-neutralizing oxygen atoms (Fig. 3 *D* and see Fig. S4).

To further test these interpretations, we ran additional MD simulations of the isolated hERG VS domain model and an R534K mutant in a hydrated POPC bilayer. Again, the R534 side chain entered the CTC in the wild-type model simulation whereas the K534 side chain did not (see Fig. S5). Inspection of the atom distributions in Fig. 3 *D* (and see Fig. S4) indicates that the pocket below the conserved Phe of the CTC is particularly favorable for a Lys side chain, with waters and acidic side chains that satisfy the spherical solvation requirements of the terminal amino group, and nonpolar side chains that interact with the aliphatic part of the side chain.

The occurrence of transitions of the R534 side chain through the CTC in the hERG model, in the absence of a change in membrane potential, indicates a relaxation from a less-stable starting structure. However, the path of the R534 side chain provides useful molecular-level insight into the nature of charge transfer in voltage sensors. How do these observations accord with broader evidence of

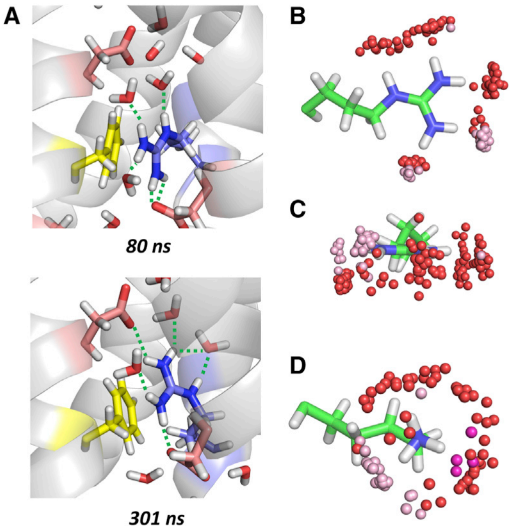

FIGURE 3 In-plane solvation of R534 guanidine in the charge transfer center during the hERG model MD (*A*). (*Dotted lines*) H-bond distances of <2.5 Å. The right-hand group consists of top-down (*B*) and end-on (*C*) views of the distribution of oxygen atoms around the side chain of hERG R534 at 20-ns intervals during MD (subunit *a*). (*D*) End-on view of equivalent atom distributions around the K302 side chain during the Kv1.2/2.1 chimera MD (subunit *c*). (*Red spheres*, water O; *pink*, Asp OD1 and OD2; *purple*:, Glu OE1 and OE2.)

charge transfer in voltage-sensitive channels in general, and hERG in particular? Studies with fluorinated analogs of aromatic side chains equivalent to F463 of hERG or F233 of the chimera indicate the absence of a significant role for cation-π interactions involving the CTC aromatic group in K^+ and Na_v channels, although a planar side chain is preferred in some cases (1,16). In hERG, F463 can be replaced by M, L, or V with small effects on channel gating (17), indicating that the hERG CTC requires only a bulky nonpolar side chain to seal the hydrophobic center of the VS and allow passage of the Arg side chain through the CTC. Both absence of requirement for cation-π interactions, and accommodation of nonplanar hydrophobic side chains in a functional hERG CTC, are broadly consistent with the interpretation that it is the poorly-hydrated nature of the Arg guanidine group above and below the molecular plane (together with its tenacious proton affinity (18)) that governs its role in carrying gating charge in voltage sensors.

While the simulations suggest that R534 may interact with D460 in the open channel state, the possibility that the extra carboxylate side chain above the CTC might facilitate gating charge transfer is seemingly inconsistent with the slow activation of hERG, although hERG D460C does activate even more slowly than the WT channel (9). However, S4 movement in hERG occurs in advance of channel opening (19), and slow gating is partly mediated by interactions involving hERG cytoplasmic domains (20); thus, slow S4 movement may not be an inherent property of the hERG voltage sensor. Recent studies show that when hERG gating is studied at very low [Ca^{2+}] (50 μM) and low [H^+] (pH 8.0), the channel is strongly sensitized in the direction of the open state; this effect is reduced in hERG D460C (and hERG D509C) (10). These observations support a role for the extra hERG Asp residues in binding Ca^{2+} (and H^+) (10), allowing the channel to be allosterically responsive to changes in pH and [Ca^{2+}]. A true comparison of a hERG model with experimental channel gating might involve studies on a channel lacking cytoplasmic domains that modulate gating, and using conditions (high pH and low [Ca^{2+}]) that leave the eag-specific Asp residues unoccupied. This could reveal the inherent current-voltage relationships and kinetics of the hERG voltage sensor.

SUPPORTING MATERIAL

Additional supplemental information, methods, and five figures are available at http://www.biophysj.org/biophysj/supplemental/S0006-3495(14)01050-9.

We are grateful to Marcelo Querino Lima Afonso for a voltage sensor simulation setup, Dr. Craig Armstrong for discussions, and a reviewer for helpful suggestions.

This work was funded by the Biotechnology and Biological Sciences Research Council (UK) through a Ph.D. studentship to C.K.C., the British Heart Foundation (grants No. PG 06/042 and No. PG 10/017), and the University of Bristol via provision of high performance computing through the Advanced Computing Research Centre.

SUPPORTING CITATIONS

References (21–27) appear in the Supporting Material.

REFERENCES and FOOTNOTES

1. Tao, X., A. Lee, …, R. MacKinnon. 2010. A gating charge transfer center in voltage sensors. *Science.* 328:67–73.
2. Catterall, W. A. 2010. Ion channel voltage sensors: structure, function, and pathophysiology. *Neuron.* 67:915–928.
3. Bezanilla, F. 2008. How membrane proteins sense voltage. *Nat. Rev. Mol. Cell Biol.* 9:323–332.
4. Chen, X., Q. Wang, …, J. Ma. 2010. Structure of the full-length *Shaker* potassium channel Kv1.2 by normal-mode-based x-ray crystallographic refinement. *Proc. Natl. Acad. Sci. USA.* 107:11352–11357.
5. Long, S. B., X. Tao, …, R. MacKinnon. 2007. Atomic structure of a voltage-dependent K^+ channel in a lipid membrane-like environment. *Nature.* 450:376–382.
6. Payandeh, J., T. Scheuer, …, W. A. Catterall. 2011. The crystal structure of a voltage-gated sodium channel. *Nature.* 475:353–358.
7. Zhang, X., W. Ren, …, N. Yan. 2012. Crystal structure of an orthologue of the NaChBac voltage-gated sodium channel. *Nature.* 486:130–134.
8. Campos, F. V., B. Chanda, …, F. Bezanilla. 2007. Two atomic constraints unambiguously position the S4 segment relative to S1 and S2 segments in the closed state of *Shaker* K channel. *Proc. Natl. Acad. Sci. USA.* 104:7904–7909.
9. Liu, J., M. Zhang, …, G.-N. Tseng. 2003. Negative charges in the transmembrane domains of the HERG K channel are involved in the activation- and deactivation-gating processes. *J. Gen. Physiol.* 121:599–614.
10. Kazmierczak, M., X. Zhang, …, T. Jegla. 2013. External pH modulates EAG superfamily K^+ channels through EAG-specific acidic residues in the voltage sensor. *J. Gen. Physiol.* 141:721–735.

11. Colenso, C. K., R. B. Sessions, ..., C. E. Dempsey. 2013. Interactions between voltage sensor and pore domains in a hERG K^+ channel model from molecular simulations and the effects of a voltage sensor mutation. *J. Chem. Inf. Model.* 53:1358–1370.

12. Schwaiger, C. S., P. Bjelkmar, ..., E. Lindahl. 2011. 3_{10}-helix conformation facilitates the transition of a voltage sensor S4 segment toward the down state. *Biophys. J.* 100:1446–1454.

13. Mason, P. E., G. W. Neilson, ..., J. M. Cruickshank. 2003. The hydration structure of guanidinium and thiocyanate ions: implications for protein stability in aqueous solution. *Proc. Natl. Acad. Sci. USA.* 100:4557–4561.

14. Mason, P. E., J. W. Brady, ..., C. E. Dempsey. 2007. The interaction of guanidinium ions with a model peptide. *Biophys. J.* 93:L04–L06.

15. Mason, P. E., C. E. Dempsey, ..., J. W. Brady. 2009. Preferential interactions of guanidinum ions with aromatic groups over aliphatic groups. *J. Am. Chem. Soc.* 131:16689–16696.

16. Pless, S. A., F. D. Elstone, ..., C. A. Ahern. 2014. Asymmetric functional contributions of acidic and aromatic side chains in sodium channel voltage-sensor domains. *J. Gen. Physiol.* 143:645–656.

17. Cheng, Y. M., C. M. Hull, ..., T. W. Claydon. 2013. Functional interactions of voltage sensor charges with an S2 hydrophobic plug in hERG channels. *J. Gen. Physiol.* 142:289–303.

18. Harms, M. J., J. L. Schlessman, ..., B. García-Moreno. 2011. Arginine residues at internal positions in a protein are always charged. *Proc. Natl. Acad. Sci. USA.* 108:18954–18959.

19. Es-Salah-Lamoureux, Z., R. Fougere, ..., D. Fedida. 2010. Fluorescence-tracking of activation gating in human ERG channels reveals rapid S4 movement and slow pore opening. *PLoS ONE.* 5:e10876.

20. Gustina, A. S., and M. C. Trudeau. 2012. HERG potassium channel regulation by the N-terminal eag domain. *Cell. Signal.* 24:1592–1598.

21. Hess, B., C. Kutzner, ..., E. Lindahl. 2008. GROMACS 4: algorithms for highly efficient, load-balanced, and scalable molecular simulations. *J. Chem. Theory Comput.* 4:435–447.

22. Wolf, M. G., M. Hoefling, ..., G. Groenhof. 2010. G_MEMBED: efficient insertion of a membrane protein into an equilibrated lipid bilayer with minimal perturbation. *J. Comput. Chem.* 31:2169–2174.

23. Jorgensen, W. L., and J. Tirado-Rives. 1988. The OPLS potential functions for proteins: energy minimizations for crystals of cyclic peptides and crambin. *J. Am. Chem. Soc.* 110:1657–1666.

24. Berger, O., O. Edholm, and F. Jähnig. 1997. Molecular dynamics simulations of a fluid bilayer of dipalmitoylphosphatidylcholine at full hydration, constant pressure, and constant temperature. *Biophys. J.* 72:2002–2013.

25. Berendsen, H. J. C., J. P. M. Postma, ..., J. Hermans. 1981. Interaction models for water in relation to protein hydration. *In* Intermolecular Forces. B. Pullman, editor. Reidel, Dordrecht, the Netherlands, pp. 331–342.

26. Bjelkmar, P., P. S. Niemelä, ..., E. Lindahl. 2009. Conformational changes and slow dynamics through microsecond polarized atomistic molecular simulation of an integral Kv1.2 ion channel. *PLOS Comput. Biol.* 5:e1000289.

27. Durdagi, S., S. Deshpande, ..., S. Y. Noskov. 2012. Modeling of open, closed, and open-inactivated states of the hERG1 channel: structural mechanisms of the state-dependent drug binding. *J. Chem. Inf. Model.* 52:2760–2774.

Voltage sensor gating charge transfer in a hERG potassium channel model

Charlotte K. Colenso,* Yang Cao,* Richard B. Sessions,*Jules C. Hancox,† and Christopher E. Dempsey*
*School of Biochemistry, and † School of Physiology and Pharmacology, University of Bristol, Bristol BS8 1TD, UK.

Supporting Material

Methods

Molecular dynamics simulation: The molecular dynamics (MD) simulation of the hERG membrane domain model in a hydrated POPC bilayer at 310 K over 0.5 μs has been described (1). The analyses here and in the main paper were made on this simulation extended to 0.85 μs.

Gromacs 4.5.3 (2) was used for all MD set up and simulation runs. We made two equivalent 0.5 – 0.55 μs MD simulations of the Kv1.2/2.1 chimera structure (the template for the hERG model) in hydrated POPC membrane patches at 310 K, one incorporating only the membrane (α) domain of the channel (0.55 μs) and one containing both the α- and cytoplasmic domains (0.50 μs). Briefly, the crystal structure coordinates (PDB: 2R9R) (3) were downloaded and the cytoplasmic (T1) domain either retained (“full”) or removed (“alpha”). Two of the K^+ ions in the selectivity filter were replaced with water molecules to yield a 0101 (K^+ = 1; water = 0) occupancy state as counted from the extracellular side of the channel. The channels were energy minimized with 1800 steps of steepest descent and conjugate gradient methods using Discover within InsightII (Accelrys; San Diego, CA, USA) and were then embedded in a POPC bilayer consisting of 576 lipid molecules in each bilayer leaflet using g-membed (4), before solvation with a 15 Å layer of water above and below the membrane. The water solution contained a number of K^+ and Cl^- ions adjusted to yield a concentration of 140 mM and to maintain overall charge-neutrality.

The simulation systems were energy minimized using 2000 steps of steepest descent and were then heated to 310 K for 5 ns of MD during which the non-hydrogen protein atoms and selectivity filter K^+ ions were restrained. Production runs were then performed for 0.85 μs (hERG model) or 0.50 - 0.55 μs (chimera models) using the OPLS all-atom forcefield (5), Berger lipid parameters (6), and the SPC water model (7). All other simulation parameters were as described in (1).

Isolated single voltage sensor simulations were made by editing the hERG model coordinate file to leave only a single voltage sensor (subunit a) which was embedded in a reduced POPC bilayer (256 lipids in each monolayer). A hERG voltage sensor R534K mutant was made by changing the R534 side chain to K534 having the same side chain rotamer as K302 in the Kv1.2/2.1 chimera structure. The systems were solvated with water and ions, energy minimized and subjected to 5 ns restrained dynamics as described above, before 200 ns production MD runs at 310 K.

Supporting Figures

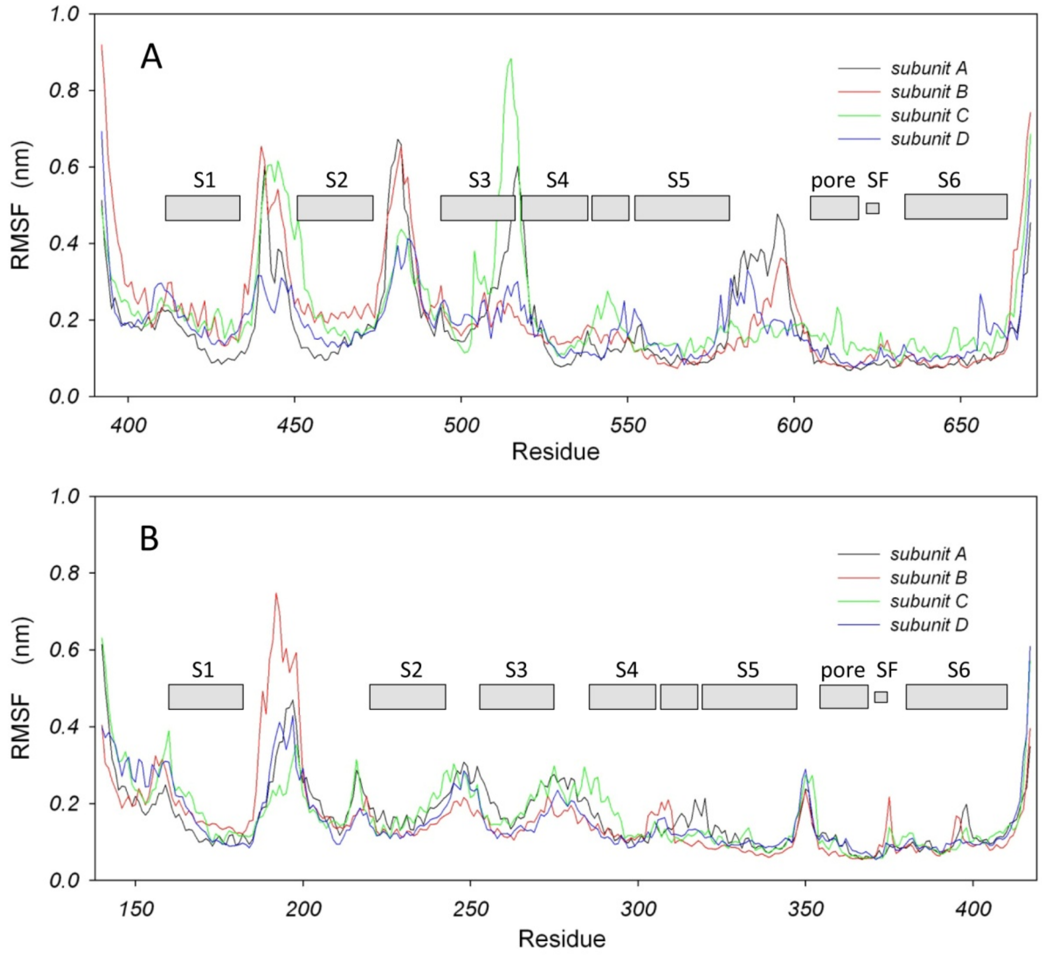

Figure S1 Root mean square fluctuation (RMSF) for Cα atoms of the hERG (A) and Kv1.2/2.1 chimera (B) membrane domain simulations in hydrated POPC membranes at 310 K. The profile of RMSF values for the chimera simulation is very similar to that described by Bjelkmar et al. (8) for a closely related (Kv1.2) K^+ channel simulation in a POPC/POPG mixed membrane at 300 K. SF = selectivity filter.

As previously found for MD simulations of membrane incorporated proteins, extramembrane loops connecting transmembrane (TM) elements often show high conformational flexibility (high RMSF) during MD (*e.g.* ref 8). This applies to the long extracellular loop between S1 and S2 in the chimera simulation (Fig. S1 B), and the extracellular loops between S1 and S2, and between S5 and the pore helix, and the cytoplasmic loop between S2 and S3 in hERG (Fig. S1 A).

The extracellular loop between S3 and S4 is truncated in hERG compared to the chimera template and this region is difficult to model onto the template chimera structure (1). Although two subunits (a and c corresponding to the black and green traces in Fig. S1 A) showed high RMSF for the C-terminal end of S3 and its linker with S4, this high flexibility did not directly affect the C-terminal end of S4 and the charge transfer centre (CTC) discussed in the main text. Of the two hERG VS subunits showing movement of the R534 side chain across the CTC (subunits a and b; see main text), one showed high RMSF in the S3-S4 linker (subunit a) and one showed low RMSF in this region (subunit b; see Fig. S1A).

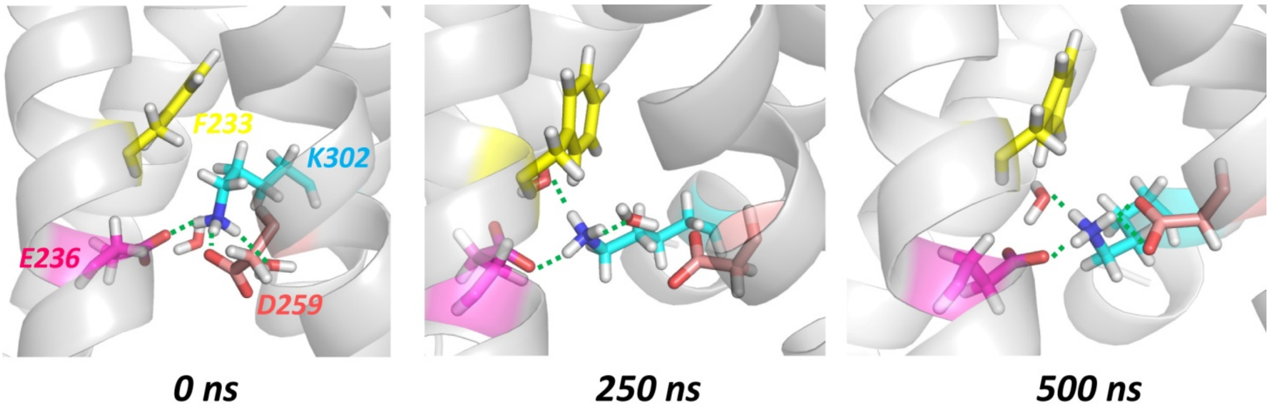

Figure S2 "Solvation" of the K302 amino group during MD simulation of the Kv1.2/2.1 chimera membrane domain. Dotted lines denote hydrogen bond distances of less than 2.5 Å.

The K302 amino group of the Kv1.2/2.1 chimera (see Fig. 1 of main text) maintained hydrogen bond interactions with carboxylate side chains and water molecules below F233 of the charge transfer centre (CTC) throughout 550 ns of MD despite fluctuations around a generally stable structure. In the example shown (subunit a) fluctuations temporarily move the K302 amino group from hydrogen bond interaction with the D259 carboxylate (e.g. at 250 ns). The stability of the Kv1.2/2.1 chimera starting structure was maintained in all 4 subunits of each of the simulations in hydrated POPC membranes in which the cytoplasmic subunit was either retained or removed (> 4 μs total simulation time).

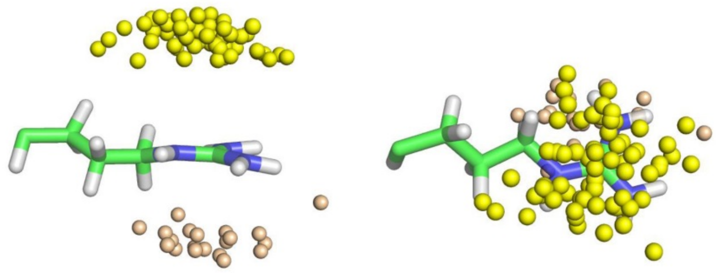

Figure S3 Distribution of non-polar carbon atoms around the R534 side chain of the WT hERG simulation as the guanidine ring moves past F463 of the CTC. Yellow spheres are side chain carbons of F463 within 4.5 Å of the guanidine CZ atom at 20 ns intervals in the first 200 ns of MD of subunits a and b (see Fig. 2 A-C main paper). Wheat-colored atoms are other hydrophobic carbons within 4.5 Å of the guanidine CZ. Left: side view; right: top-down view.

The solvation-deficient faces of the guanidine side chain (see Fig. 3 B,C of main paper) can interact with solvation-deficient surfaces of non-polar amino acids as previously described (9, 10). In this case the R534 side chain slides between the F463 side chain (yellow spheres in Fig. S3) and other non-polar amino acids (largely side chain carbons of I500 and A504 on the S3 helix).

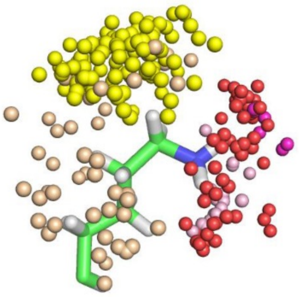

Figure S4 Atom distributions around the K302 side chain of the Kv1.2/2.1 chimera VS (see Fig. 1 and Fig. S2) at 20 ns intervals during MD (20-540 ns; subunit c). Yellow spheres: F233 side chain carbons; wheat: other non-polar C atoms; red: water O atoms; pink: Asp carboxylate O atoms; purple: Glu carboxylate O atoms. This is the same compilation as shown in Fig. 3D of the main paper but rotated 180 ^{o} around z and y (x,y is in the plane of the page), and with non-polar C-atoms within 4.5 A of Cδ, Cε and Nz added.

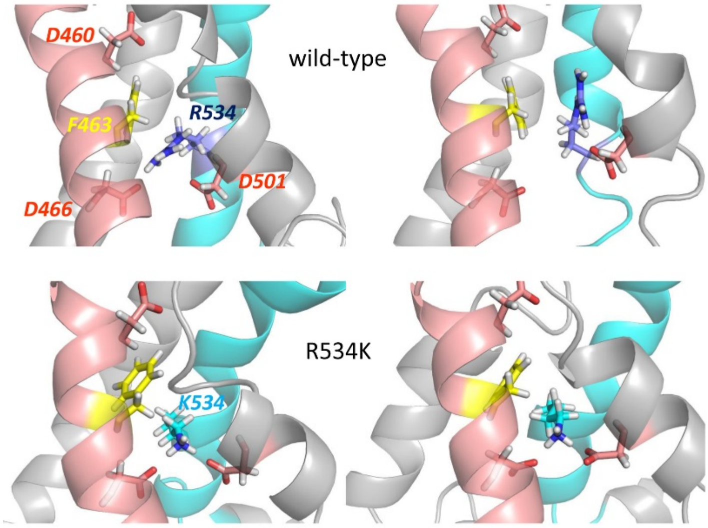

Figure S5 Starting (left) and final (200 ns; right) conformation of isolated hERG wild-type (top) and R534K model (bottom) voltage sensor simulations in a solvated POPC bilayer patch at 310 K. The simulations were run as described under Methods.

The cavity below the conserved Phe residue (F233 of the Kv1.2/2.1 chimera; F463 of hERG) may constitute a “trap” for the Lys side chain. As indicated in the Fig. S2 legend, the K302 side chain of the chimera simulation did not move from this cavity during a total of 4 μs of MD (4 subunits each of the chimera simulation with and without the cytoplasmic domain). Fig. S4 illustrates that the environment of this cavity conforms to the heterogeneous nature of the Lys side chain with non-polar interactions between chimera residues (F233: yellow spheres; I173, A262 and I263: wheat spheres in Fig. S4) and the non-polar part of the K302 side chain, with waters and carboxylate O atoms of E236 and D259 solvating the charge focused on the end of the side chain.

These interpretations are supported by additional simulations of the isolated wild-type hERG VS domain and an R534K “mutant” in a hydrated POPC bilayer. The R534 side chain guanidine moved into the non-polar gap of the CTC in the wild-type simulation (Fig. S5 top), while the K534 side chain of the mutant remained fixed in the cavity below the F463 side chain of the CTC. An additional 200 ns R534K mutant run in which the mutant VS was independently re-embedded in the POPC bilayer before MD also resulted in a stable retention of the K534 side chain in the pocket below F463 (not shown).

We note that the hERG membrane domain open channel model of Durdagi et al. (11) has a similar set of VS charge interactions as in our wild-type hERG model. However MD simulations in that study were of 7.5

ns duration, limiting the possibility of observing transitions on the ~ 100 ns timescale and precluding direct comparison with the VS transitions described here.

Supporting References

1. Colenso, C. K., R. B. Sessions, Y.-H. Zhang, J. C. Hancox and C. E. Dempsey. 2013. Interactions between voltage sensor and pore domain in a hERG K^+ channel model from molecular simulations and the effects of a voltage sensor mutation. *J. Chem. Inf. Model.* 53: 1358-1370.

2. Hess, B., C. Kutzner, D. van der Spoel and E. Lindahl. 2008. GROMACS 4: Algorithms for highly efficient, load-balanced, and scalable molecular simulations. *J. Chem. Theory Comput.* 4: 435-447.

3. Long, S. B., X. Tao, E. B. Campbell and R. Mackinnon. 2007. Atomic structure of a voltage-dependent K+ channel in a lipid membrane-like environment. *Nature* 450: 376-382.

4. Wolf, M. G., M. Hoefling, C. Aponte-Santamaria, H. Grubmuller and G. Groenhof. 2010. g_membed: Efficient insertion of a membrane protein into an equilibrated lipid bilayer with minimal perturbation*. J. Comput. Chem.* 31: 2169-2174.

5. Jorgensen W. L. and J. Tirado-Rives. 1988. The OPLS potential function for proteins – energy minimizations for crystals of cyclic-peptides and crambin. *J. Am. Chem. Soc.* **110**, 1657-1666.

6. Berger, O., O. Edholm and F. Jahnig. 1997. Molecular dynamics simulations of a fluid bilayer of dipalmitoylphosphatidylcholine at full hydration, constant pressure, and constant temperature. *Biophys. J.* **72**, 2002-2013.

7. Berendsen, H., J. P. M. Postma, W. F. van Gunsteren and J. Hermans. 1981. Interaction models for water in relation to protein hydration. In *Intermolecular Forces*; Pullman, B. Ed.; Reidel: Dordrecht; pp 331-342.

8. Bjelkmar, P., P. S. Niemelä, I. Vattulainen and E. Lindahl. 2009. Conformational changes and slow dynamics through microsecond polarized atomistic molecular simulation of an integral Kv1.2 ion channel. *PLoS Comp. Biol.* e1000289.

9. Mason, P. E., J. W. Brady, G. W. Neilson and C. E. Dempsey. 2007. The interaction of guanidinium ions with a model peptide. *Biophys. J.* 93: L04-L06.

10. Mason, P. E., C. E. Dempsey, G. W. Neilson, S. R. Kline and J. W. Brady. 2009. Preferential interaction of guanidinium ions with aromatic groups over aliphatic groups. *J. Am. Chem. Soc.* 131: 16680-16696.

11. Durdagi, S., S. Deshpande, H. J. Duff and S. Y. Noskov. 2012. Modeling of open, closed and open-inactivated states of the hERG1 channel: structural mechanisms of the state-dependence of drug binding. *J. Chem. Inf. Model.* 52: 2760-2774.
